# Divergent Tissue and Circulatory Expression of miR-10a in Canine Hepatocellular Carcinoma: Comparative Insights from Human HCC

**DOI:** 10.3390/cimb47110950

**Published:** 2025-11-15

**Authors:** Most Shumi Akhter Shathi, Mohammad Arif, Nobuhiro Nozaki, Yutaro Ide, Yoshiyuki Akiyama, Shaohsu Wang, Masashi Takahashi, Naoki Miura

**Affiliations:** 1 Joint Graduate School of Veterinary Medicine, Kagoshima University, Kagoshima 890-0065, Japanmdarif38515@bau.edu.bd (M.A.);; 2 Department of Microbiology and Hygiene, Bangladesh Agricultural University, Mymensingh 2202, Bangladesh; 3 Veterinary Teaching Hospital, Joint Faculty of Veterinary Medicine, Kagoshima University, Kagoshima 890-0065, Japan

**Keywords:** canine hepatocellular carcinoma, miR-10a, exosome, biomarker, next generation sequencing, comparative oncology

## Abstract

Canine hepatocellular carcinoma (HCC), the most common primary liver malignancy in dogs, shares many clinicopathological and molecular similarities with human HCC. However, its molecular characteristics remain insufficiently defined, and reliable diagnostic biomarkers are lacking. Elucidating dysregulated microRNAs (miRNAs) may aid in both disease characterization and comparative oncology research. Small RNA sequencing datasets from canine HCC were analyzed to identify significantly dysregulated miRNAs with high expression and biomarker potential. The top candidate was validated in clinical tissues, cell lines, patient’s plasma and plasma exosomes using RT-qPCR. Comparative analyses were conducted using human HCC datasets (TCGA and GEO), followed by target prediction and functional enrichment to identify conserved molecular pathways. Among the 59 differentially expressed miRNAs, cfa-miR-10a showed the highest average expression level and yet was significantly downregulated in canine HCC tissues. RT-qPCR confirmed reduced expression of cfa-miR-10a in canine HCC tissues, whereas plasma exosomes showed significant enrichment, demonstrating excellent diagnostic performance (AUC = 0.94). The mature sequence of cfa-miR-10a is highly conserved with hsa-miR-10a-5p. TCGA datasets confirmed downregulation of hsa-miR-10a-5p in HCC tissues, whereas a GEO dataset showed no significant change in serum exosome levels. Target prediction and functional annotation identified 59 overlapping genes, with the Proteoglycans in cancer pathways being conserved in both species, mediated by ACTG1, SDC1, FRS2, and WNT9B. Collectively, these findings demonstrate distinct intra-tumoral and exosomal expression pattern of miR-10a in canine HCC and support its potential as a non-invasive biomarker with translational relevance.

## 1. Introduction

Hepatocellular carcinoma (HCC) is the most common primary liver cancer in both humans and dogs [1,2], representing a major cause of cancer-related deaths worldwide. In people, HCC ranks as the sixth most commonly diagnosed cancer [3] and the third leading cause of cancer-related mortality [4], with incidence continuing to rise due to chronic hepatitis, cirrhosis, and metabolic liver disease [5]. Despite advances in imaging and surgical approaches, many patients are still only being diagnosed at an advanced stage, when treatment options remain limited [6,7]. In veterinary medicine, HCC is the most frequent primary liver tumor in dogs, accounting for about 77% of cases [8], and is often diagnosed incidentally during abdominal imaging or at an advanced stage when clinical signs become apparent [9].

Cell lines have long been used as in vitro models to study the biological characteristics of cancer cells. Although they offer several advantages, such models lack the native tumor microenvironment and may undergo adaptive changes during prolonged culture. These limitations reduce their ability to accurately represent in vivo tumor biology and circulating biomarker profiles [10]. Murine models remain invaluable for in vivo cancer research; however, their relatively short lifespan, genomic instability, restricted immune interactions, and limited tumor heterogeneity often constrain their predictive relevance to human disease. Consequently, there is an increasing need for spontaneous animal models that more closely mirror the complexity of human cancers. Companion dogs provide such a model. Their naturally occurring tumors closely resemble human cancers in terms of histopathology, genetics, molecular signaling pathways, and therapeutic responses [10]. Moreover, dogs share many environmental risk factors with humans, serving as important sentinels for disease. Their high degree of genetic similarity to humans also facilitates the identification of genes associated with tumor development [11]. Together, these factors underscore the translational importance of dogs as a model for human cancer, providing a biologically and genetically relevant system for studying tumor development and evaluating novel diagnostic and therapeutic strategies.

Canine HCC shares many histopathological, molecular, and clinical features with human HCC and is increasingly regarded as a valuable potential spontaneous model of the human HCC [12,13,14,15]. Human HCC is frequently linked to chronic viral hepatitis, cirrhosis, alcohol, or metabolic liver disease, whereas canine HCC more often arises in the absence of chronic viral infection or cirrhosis. This distinction makes the canine model particularly useful for investigating tumor-intrinsic genetic and microenvironmental drivers of hepatocarcinogenesis that are independent of background chronic liver injury [16]. Molecular characterization of canine HCC could thus provide translational insights into hepatocarcinogenesis.

MicroRNAs (miRNAs) are small, noncoding RNAs that regulate gene expression at the post-transcriptional level by binding to complementary sequences in target mRNAs. Crucially, they help to regulate diverse biological processes, including cell proliferation, apoptosis, and migration, and their dysregulation contributes to the onset and progression of numerous cancers [17]. Owing to their stability in formalin-fixed tissues and biofluids, miRNAs have emerged as promising biomarkers for cancer diagnosis, prognosis, and therapeutic monitoring [17,18].

Circulating miRNAs have drawn considerable attention as liquid biopsy markers, offering a noninvasive approach for tumor detection and monitoring. Many are encapsulated within extracellular vesicles (EVs), especially exosomes, which protect them from enzymatic degradation and facilitate their transport in the bloodstream [19]. Tumor cells can selectively package specific miRNAs into exosomes, thereby modulating the tumor microenvironment and systemic signaling [20]. Notably, the expression of circulatory miRNAs does not always correspond to that in tissue counterparts [21,22]. This discrepancy may arise from passive release due to necrosis or apoptosis versus active secretion [23], suggesting that circulating miRNAs represent an interesting avenue to explore for biomarker potential, and may ultimately provide clinicians with additional insight into cancer progression beyond tissue-level miRNA expression.

Among the cancer-associated miRNAs, miR-10a has gained attention due to its context-dependent roles. It can reportedly function either as a tumor suppressor or an oncomiR, depending on the tissue type [24]. In several malignancies, miR-10a functions as a tumor suppressor, where its downregulation in tumor tissues is linked to more aggressive phenotypes [25,26,27]. Conversely, as an overexpressed oncomiR in certain malignancies, it has been reported to promote cancer cell proliferation and metastasis [28,29,30], highlighting the complexity of its regulation and function. In human HCC, most studies have demonstrated significant downregulation of miR-10a in tumor tissues relative to adjacent normal liver [25,31,32,33], a pattern thought to drive malignant progression through derepression of oncogenic pathways. Consistently, circulating analyses have shown reduced plasma levels of miR-10a in HCC patients [25], reinforcing its potential role as a tumor-suppressive miRNA in the human liver cancer context.

In contrast, little is known about the role of miR-10a in canine HCC. To date, there are only a few reports on transcriptomic [14] and small non-coding RNA [34,35] research in canine HCC in general, and there is a paucity of data on miR-10a specifically. Given the potential for dogs to act as a spontaneous disease model for human HCC, there is a need to profile miR-10a expression in both HCC tumor tissue and blood circulation, as part of the assessments of the translational potential of the relevant canine model. Therefore, in this study, we aimed to characterize the expression profile of miR-10a in canine HCC tissues and plasma exosomes and to contextualize these findings with available human data, thereby contributing to the development of miRNAs as diagnostic biomarkers in both veterinary and human oncology.

## 2. Materials and Methods

### 2.1. Experimental Design

In this study, we set out to profile the expression of miR-10a in HCC clinical tissues and circulatory compartments (including plasma and plasma exosomes) to assess its biomarker potential in canine HCC, using sequence data that had been previously analyzed to characterize the miRNA transcriptome [35]. We re-analyzed raw small RNA sequencing files (FASTQ) from canine HCC and normal liver tissues with an updated miRNA database (miRBase v22.1) and the most recent version of the relevant analysis software (CLC Genomics Workbench v24.0, Qiagen, Germany) to incorporate recent annotation updates. Our goal was to systematically identify the most abundantly expressed and dysregulated miRNA candidate under the revised analysis framework, validate it in canine HCC tissues and circulating compartments to assess its biomarker potential, and perform a comparative assessment in the context of human HCC.

### 2.2. Clinical Samples

Fourteen liver tissue samples (*n* = 14) and nine blood samples (*n* = 9) from individual canine HCC patients were included in this study. Samples were collected from dogs undergoing surgical treatment at the Veterinary Teaching Hospital of Kagoshima University (KUVTH) or affiliated veterinary clinics. Canine HCC was diagnosed histopathologically by specialized pathologists at the respective institutions. Samples showing histopathological confirmation along with the absence of other concurrent neoplastic or severe systemic diseases were included in this study, while samples with unclear pathological diagnosis and degraded RNA quality were excluded from this study. Informed consent was obtained from each dog owner prior to sample collection. The study population comprised dogs of various breeds (including Beagle, Shiba, Miniature Schnauzer, Toy Poddle, Pomeranian, Yorkshire Terrier and mixed breeds), aged between 4 and 14 years, with both males and females represented. The clinical and physiological parameters (e.g., blood cells, glucose, creatinine, serum ALT, ALP, and urea levels, where available) are summarized in Table S1.

For controls, liver tissues from six healthy adult laboratory Beagle dogs (*n* = 6) were obtained from Shin Nippon Biomedical Laboratories, Ltd. (Tokyo, Japan) [36]. These animals were maintained as healthy dogs under controlled laboratory conditions for unrelated experimental purposes and were sacrificed according to the institutional protocols approved by the Association for Assessment and Accreditation of Laboratory Animal Care (AAALAC). Liver samples were collected aseptically at necropsy, immediately immersed in RNAlater to stabilize RNA, and shipped to Kagoshima University under temperature-controlled conditions for molecular analysis. Blood samples (*n* = 7) from clinically healthy dogs free of tumors were collected from Chuou Aiken Animal Hospital (Kagoshima, Japan).

Immediately after collection, clinical tissue samples were stored in RNAlater (Thermo Fisher Scientific, Vilnius, Lithuania) and transported to the laboratory under proper cold-chain conditions. To obtain plasma from healthy dog and clinical patients, blood samples were first centrifuged (TOMY MX-105, Tokyo, Japan) at 3000× *g* for 10 min, followed by a second centrifugation at 16,000× *g* for 20 min to further purify the plasma. The supernatant was carefully collected to avoid contamination with the pellet. All tissue and plasma samples were stored at −80 °C until further analysis.

For next-generation sequencing (NGS), three representative tissue samples from each group (HCC and normal liver) were selected based on high RNA integrity number (RIN > 8.0), and histological uniformity to ensure high-quality sequencing data. The remaining samples were used for RT-qPCR validation. All animal associated procedures were approved by the animal ethics committee of KUVTH (Approval No. 220001) and were conducted in accordance with KUVTH regulations and guidelines.

### 2.3. Cell Lines and Cell Culture

The present study utilized two canine HCC cell lines, 95-1044 (fast-proliferating) and 95-0112 (slow-proliferating), which were previously characterized in other studies [36,37]. The cell lines were stored in liquid nitrogen with a CultureSure freezing medium (Wako Pure Chemical 88 Industries, Ltd., Osaka, Japan) and cultured as previously described [36] in a CO_2_ incubator (HeracellTM CO_2_ incubator, Thermo Fisher Scientific). Briefly, Dulbecco’s Modified Eagle’s Medium containing 5% fetal bovine serum (Thermo Fisher Scientific, Waltham, MA, USA), 5% L-glutamine (Sigma-Aldrich, St. Louis, MO, USA), and 3.5 μg/mL spectinomycin (Sigma-Aldrich, St. Louis, MO, USA) were used. Culture plates were incubated at 37 °C in 5% CO_2_ and cell lysates were collected after 48 h. Upon reaching confluency, cells were washed with PBS and lysed directly on culture plates using the lysis buffer supplied with the mirVana miRNA Isolation Kit (Thermo Fisher Scientific, Waltham, MA, USA). The resulting lysates were passed several times through a 23-gauge needle to reduce viscosity and facilitate efficient nucleic acid extraction. Following collection, lysates were either processed for RNA isolation or stored at −80 °C for further use.

### 2.4. Exosome Isolation

Small extracellular vesicles (sEVs, often known as exosomes) were isolated from plasma using the Total Exosome Isolation (from plasma) Kit (Thermo Fisher Scientific, Waltham, MA, USA) according to the manufacturer’s instructions. Briefly, 300 µL of plasma was mixed with 150 µL of 1X PBS, followed by the addition of 90 µL of exosome precipitation reagent. The mixture was vortexed thoroughly and incubated at room temperature for 10 min. Exosomes were then pelleted by centrifugation (TOMY MX-105, Tokyo, Japan) at 10,000× *g* for 5 min at room temperature. Supernatant was carefully discarded without disturbing the exosome pellet, which was then suspended in 150 µL of 1X PBS and stored at −80 °C for further analysis.

### 2.5. RNA Extraction and Next Generation Sequencing (NGS) of Small RNAs

Total RNA from cell lysates and tissues was extracted using a mirVana™ miRNA Isolation Kit (Thermo Fisher Scientific, Waltham, MA, USA), and from plasma and plasma exosome using mirVana PARIS Kit (Thermo Fisher Scientific, Waltham, MA, USA) and Total Exosome RNA and Protein Isolation Kit (Thermo Fisher Scientific, Waltham, MA, USA), respectively. For RNA extraction, cell lysates or clinical tissue samples were mixed with equal volume of lysis buffer, while the plasma samples with 2X denaturation solution. The mixture was further added with miRNA Homogenate Additive (one tenth of total volume), and then incubated at 4 °C for 10 min. After that, Acid-phenol/chloroform (Ambion^®^, Thermo Fisher Scientific, Waltham, MA, USA) was added at an equal volume of initial samples, vortexed thoroughly and then centrifuged at 15,000× *g* for 5 min at room temperature. After centrifugation, the upper aqueous phase was carefully collected, and its volume was recorded. After that, 1.25 volume of 99.9% ethanol was then added to the collected aqueous phase and mixed gently. The mixture was then centrifuged using spin column to trap the RNA particle on the filter paper, followed by several washing with buffers. Finally, pre-heated elution solution at 95 °C was utilized to elute total RNA and its concentration was measured using the NanoDrop 2000c spectrophotometer (Thermo Fisher Scientific). RNA quality and integrity were assessed using an Agilent 2100 Bioanalyzer (G2939BA, Agilent Technologies, Santa Clara, CA, USA).

Small RNA sequencing was performed on three HCC tissues and three normal liver tissues by NGS as described earlier [35]. Briefly, total RNA from the respective samples was sent to Hokkaido System Science Co. Ltd., (Sapporo, Hokkaido, Japan) to perform high throughput sequencing. Small RNA libraries were prepared using the TruSeq Small RNA Library Preparation kit from 1 µg of total RNA following the manufacturer’s instructions (Illumina, San Diego, CA, USA), followed by adapters (5′ and 3′) ligation to the small RNAs, cDNA generation, and subsequent amplification. The amplified cDNA was then gel purified and sequenced through Illumina/Hiseq2500 sequencing.

### 2.6. Bioinformatic Analysis

Sequence reads were analyzed using the CLC Genomics Workbench v24.0 (Qiagen, Hilden, Germany, https://digitalinsights.qiagen.com, accessed on 11 March 2025) in accordance with the developer’s instructions. The analysis workflow included adapter trimming, quality control, and removal of ambiguous reads. After preprocessing, reads were annotated against miRBase v22.1 (https://mirbase.org/download/CURRENT/, accessed on 11 March 2025) with *Canis lupus familiaris* and *Homo sapiens* reference species. Differentially expressed miRNAs (DE miRNAs) were identified using a negative binomial model with Benjamini–Hochberg FDR correction. DE miRNAs were filtered by |fold change| > 2, FDR-adjusted *p* < 0.05, and a maximum group mean > 10. Principal component analysis (PCA) and heatmap plots were generated in CLC Genomics Workbench to assess sample clustering and overall expression patterns, whereas volcano plots were specifically used to visualize the differentially expressed annotated miRNAs.

### 2.7. Real-Time Quantitative PCR

Relative expression of cfa-miR-10a in clinical tissues, cell lines, plasma and plasma exosomes were assessed using real time quantitative PCR (RT-qPCR), as described previously [38]. Briefly, 1.25 μL (2 ng/μL for tissues or cells) of total RNA was reverse transcribed to cDNA in a T100 thermal cycler (Bio-Rad) using the TaqMan MicroRNA Reverse Transcription Kit (Thermo Fisher Scientific) in accordance with the manufacturer’s protocol. qPCR was performed using a TaqMan Fast Advanced Master Mix kit and a Quant Studio 3 real-time PCR system (Thermo Fisher Scientific). The thermocycling conditions were set as 50 °C for 2 min, 95 °C for 20 s; followed by 40 cycles of denaturation at 95 °C for 1 s and annealing/extension at 60 °C for 20 s, following the manufacturer’s protocol. All experimental data were reproduced at least twice. The 2^−ΔΔCT^ method was used to evaluate expression level, that is normalized using internal controls-RNU6B (TaqMan MicroRNA assays, ID: 001093, Thermo Fisher Scientific) for tissues and cells, miR-16 (ID: 000391) for plasma, and miR-186 (ID: 002285) for EV samples [38].

### 2.8. Western Blotting

Isolated exosomes were lysed on ice in RIPA Buffer (Thermo Fisher Scientific) and supplemented with Halt™ Protease Inhibitor Cocktail (Thermo Fisher Scientific) with periodic vortexing to ensure complete lysis. A total of 10 µL of protein lysate was mixed with the equal volume of 2X Laemmli Sample Buffer (Bio-Rad), heated at 95 °C, and separated on a 12% sodium dodecyl sulfate polyacrylamide gel (SDS-PAGE) alongside Precision Plus Protein™ Standards (Bio-Rad Laboratories, Hercules, CA, USA) as molecular weight markers. Proteins were then transferred onto PVDF membranes (Millipore) using Trans-Blot Turbo Transfer System (Bio-Rad). Membranes were blocked with 5% non-fat milk in TBST at room temperature for 2 h and incubated overnight at 4 °C with the following primary antibodies: anti-TSG101 (1:1000 dilution, ARP37310-T100, Aviva System Biology, San Diego, CA, USA) and anti-CD9 (1:1000 dilution, SM1065PS, OriGene Technologies GmbH, Herford, Germany). Membranes were then washed three times with TBST for 10 min each and incubated at room temperature for 2 h with HRP-conjugated secondary antibodies: anti-rabbit IgG (1:25,000 for TSG101; Jackson Immuno Research, West Grove, PA, USA) or anti-mouse IgG (1:50,000 for CD9; Jackson Immuno Research). Following additional washes with TBST, protein bands were visualized using AmershamTM ECL Prime Western Blotting Detection Reagent (Cytiva, GE Healthcare, Buckinghamshire, UK, Cat# RPN2232) and imaged with a LuminoGraph I imager (ATTO corporation, Tokyo, Japan).

### 2.9. Human Database Analysis

Expression status of hsa-miR-10a-5p was assessed in human HCC using online TCGA databases (UALCAN, available at: https://ualcan.path.uab.edu/, accessed on 23 March 2025; ENCORI, available at https://rnasysu.com/encori/, accessed on 23 March 2025), whereas GEPIA (http://gepia2.cancer-pku.cn/, accessed on 23 March 2025) and UALCAN were employed to analyze the expression of target genes. To assess the expression status of target miRNA in plasma exosomal compartment, a publicly available human HCC dataset (GSE83977) was retrieved and analyzed using the CLC Genomics Workbench v24.0 (Qiagen, Germany, https://digitalinsights.qiagen.com, accessed on 11 March 2025), following the same procedures applied for the analysis of canine HCC datasets.

### 2.10. Target Gene Prediction and Cross-Species Functional Annotation

The potential target genes of canine miR-10a (cfa-miR-10a) and its conserved human counterpart (hsa-miR-10a-5p) were predicted using two different algorithms: TargetScan v8.0 (http://www.targetscan.org/, accessed on 5 May 2025) and miRDB (http://mirdb.org/cgi-bin/search.cgi, accessed on 5 May 2025). Species specific target genes were retrieved separately for dogs and humans. To increase confidence in the predictions, only genes identified by both algorithms were obtained for subsequent analysis. The resulting miRNA-target genes interaction network was constructed using Cytoscape v3.10.3. Functional enrichment analyses, including Gene Ontology (GO) terms and Kyoto Encyclopedia of Genes and Genomes (KEGG) pathways, were then performed using the DAVID Bioinformatics Resources (https://davidbioinformatics.nih.gov, accessed on 7 May 2025) for each species-specific gene set to explore the biological relevance of miR-10a in canine and human HCC.

### 2.11. Statistical Analysis

Expression levels of cfa-miR-10a in HCC clinical tissues and normal liver tissues was compared using Student’s *t*-test. At the beginning, normality of the data was assessed using the Shapiro–Wilk test. For normally distributed data, an unpaired Student’s *t*-test was applied. If unequal variances were detected by Levene’s test, Welch’s correction was used instead. For non-normally distributed data, the non-parametric Mann–Whitney U-test was performed. Receiver Operating Characteristic (ROC) curve was generated to evaluate the diagnostic potential of the miRNA. The area under the curve (AUC) was computed, and 95% confidence intervals for sensitivity and specificity were estimated using the Wilson–Brown method. *p*-values < 0.05 were considered statistically significant, and *p*-values < 0.01 as highly significant (* *p* < 0.05; ** *p* < 0.01, *** *p* < 0.001). All statistical analyses and visualizations were performed using GraphPad Prism v8.0 (GraphPad Software, San Diego, CA, USA).

### 2.12. Use of Artificial Intelligence Tools

We employed ChatGPT-4o (OpenAI) for English grammar correction and sentence structure refinement. All AI-assisted outputs were critically reviewed, verified, and revised by the authors to ensure their appropriateness. No content related to study design, data analysis, or interpretation of results was generated by artificial intelligence.

## 3. Results

### 3.1. miR-10a Is Strongly Downregulated in Canine HCC

NGS analysis of small RNA reads from HCC vs. normal liver tissues revealed that cfa-miR-10a was the most abundantly expressed and significantly downregulated miRNA (fold change: −5.37, FDR *p*-value: 9.91 × 10^−3^, and maximum group mean: 1,629,083.33) in canine HCC (Supplementary Table S2). Interestingly, it was not detected in the earlier miRNA transcriptome study [35]. Given its marked downregulation in HCC tissues, we selected cfa-miR-10a for validation in downstream RT-qPCR experiments.

### 3.2. RT-qPCR Validation Confirms the Discrepant Expression of cfa-miR-10a in Canine HCC

**Figure 1:**
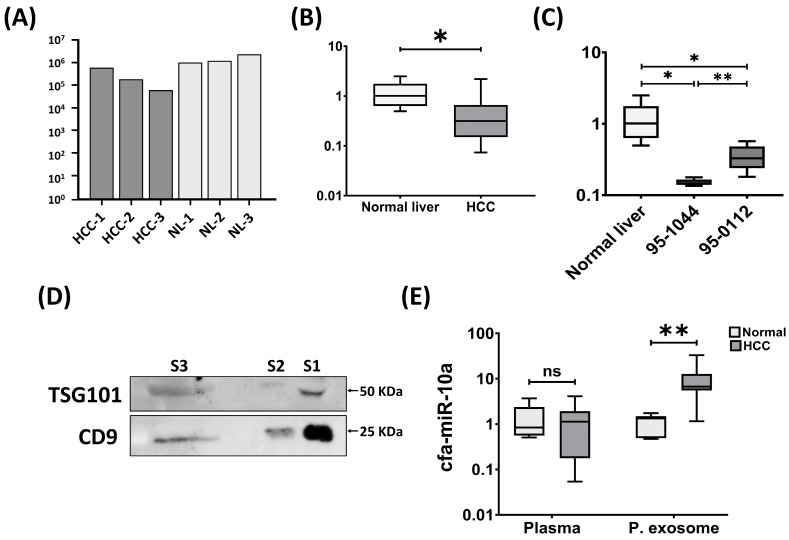
Expression pattern of cfa-miR-10a across multiple sample types. (**A**) Relative expression of cfa-miR-10a in NGS data from canine HCC tissues (HCC, *n* = 3) and normal liver tissues (NL, *n* = 3). (**B**) RT-qPCR validation of cfa-miR-10a expression in clinical samples (normal liver, *n* = 6; HCC, *n* = 14). (**C**) Relative expression of cfa-miR-10a in canine HCC cell lines compared with normal liver tissues using RT-qPCR (normal liver, *n* = 6; 95-1044, *n* = 6; 95-0112, *n* = 6). (**D**) Western blot confirmation of exosomal markers CD9 and TSG101 in plasma-derived exosomes. S1 and S2 represent exosome samples isolated from healthy dogs, while S3 corresponds to an exosome sample from a canine HCC case. Molecular weight markers (kDa) are indicated on the right. (**E**) RT-qPCR analysis of cfa-miR-10a in plasma and plasma exosomes (healthy plasma, *n* = 5; HCC plasma, *n* = 7; healthy exosome, *n* = 7; HCC exosome, *n* = 9). RNU6B, miR-16 and miR-186 were used as internal controls for tissues and cells, plasma, and exosomes, respectively, to normalize the expression of cfa-miR-10a. Normality of data distribution was assessed using the Shapiro–Wilk test. For normally distributed data, unpaired Student’s *t*-tests were performed, with Welch’s correction applied when variances were unequal (Levene’s test). For non-normally distributed data, the Mann–Whitney U-test was used. Statistical significance was set at *p* < 0.05 (* *p* < 0.05, ** *p* < 0.01 and ns—not significant). Bar graph, and box and whisker plots were generated using GraphPad Prism v8.00.

To validate the reliability of NGS findings, the expression of cfa-miR-10a was examined in clinical tissue samples. Consistent with the sequencing data, cfa-miR-10a expression significantly decreased in HCC tissues (Figure 1A,B). To further assess its expression in vitro, we examined two canine HCC cell lines: 95-1044, characterized by relatively rapid proliferation, and 95-0112, a slower proliferating line. In the absence of a normal hepatocyte reference cell line, expression levels were compared against normal liver tissues. cfa-miR-10a was significantly downregulated in both HCC cell lines, with more pronounced reduction in the highly proliferative 95-1044 cells (Figure 1C). These findings suggest that loss of cfa-miR-10a may be linked to proliferative capacity in canine HCC cells.

To evaluate the biomarker potential of cfa-miR-10a in plasma and plasma-derived exosomes, plasma was first separated from whole blood and exosomes were subsequently isolated and validated by Western blotting using two distinct protein markers, CD9 and TSG101 (Figure 1D and Supplementary Figure S1). The expression of cfa-miR-10a was then examined in plasma and plasma-derived exosome from canine HCC patients and healthy controls using RT-qPCR. Interestingly, cfa-miR-10a was significantly upregulated in plasma exosomes (*p* < 0.01), whereas no significant change was observed in plasma (*p* > 0.05) (Figure 1E).

**Figure 2:**
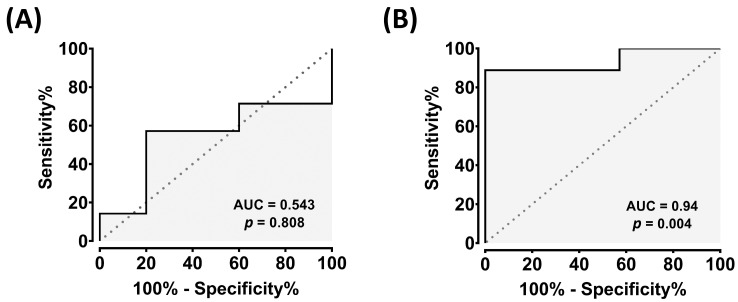
Diagnostic performance of cfa-miR-10a in plasma and plasma exosomes. (**A**) Receiver operating characteristic (ROC) curve of cfa-miR-10a in plasma (AUC = 0.54, *p* = 0.81). (**B**) ROC curve of cfa-miR-10a in plasma exosomes (AUC = 0.94, *p* = 0.004). The area under the curve (AUC) was calculated, and 95% confidence intervals for sensitivity and specificity were estimated using the Wilson–Brown method. *p*-values < 0.05 were considered as significant. ROC curves were generated using GraphPad Prism v8.00.

Consistently, receiver operating characteristic (ROC) analysis demonstrated excellent diagnostic performance for exosomal cfa-miR-10a, with areas under the curve (AUC) of 0.94 (*p* < 0.01), while plasma levels showed poor discrimination (AUC = 0.54, *p* > 0.05) [Figure 2]. Together, these results reveal a marked discrepancy between tissue and circulating exosomal expression of cfa-miR-10a, underscoring its potential as a noninvasive biomarker in canine HCC.

### 3.3. miR-10a Ortholog Is Evolutionarily Conserved and Downregulated in Human HCC

**Figure 3:**
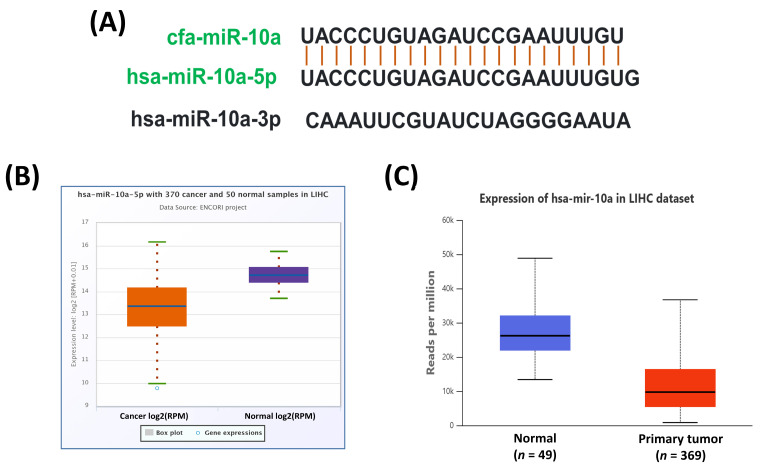
Sequence conservation of cfa-miR-10a and expression of its human counterpart in HCC. (**A**) Sequence alignment of mature cfa-miR-10a with its conserved human homolog hsa-miR-10a-5p. (**B**,**C**) Expression of hsa-miR-10a-5p in human HCC based on TCGA data analyzed through the ENCORI (**B**) and UALCAN (**C**) databases.

To examine the evolutionary conservation of miR-10a, we compared its mature sequence with the human orthologs (hsa-miR-10a-5p and hsa-miR-10a-3p) using the miRBase database. This analysis confirmed mapping between cfa-miR-10a and hsa-miR-10a-5p, suggesting potentially conserved functional relevance across species (Figure 3A). We then investigated the expression of hsa-miR-10a-5p in human HCC using TCGA datasets through ENCORI and UALCAN, which consistently showed significant downregulation of hsa-miR-10a-5p in HCC (Figure 3B,C).

### 3.4. Public Exosomal Dataset Analysis Reveals Limited Association of miR-10a with Human HCC

We analyzed a publicly available serum exosomal miRNA dataset (GSE83977) generated from human HCC patients to investigate the expression status of miR-10a. Following differential expression analysis using the same filtering criteria applied to the canine HCC sequencing dataset, we identified a total of 40 significantly differentially expressed miRNAs (Supplementary Table S3). The overall distribution and clustering patterns have been illustrated using principal component analysis (PCA), a volcano plot, and a heatmap (Supplementary Figure S2). Notably, hsa-miR-10a-5p (maximum group mean: 45,460.18, fold change: −1.22, *p* = 0.3) was downregulated but not significantly altered, highlighting its limited association with human HCC.

### 3.5. Conserved Proteoglycan-Related Signaling in Cross-Species HCC Progression

**Figure 4:**
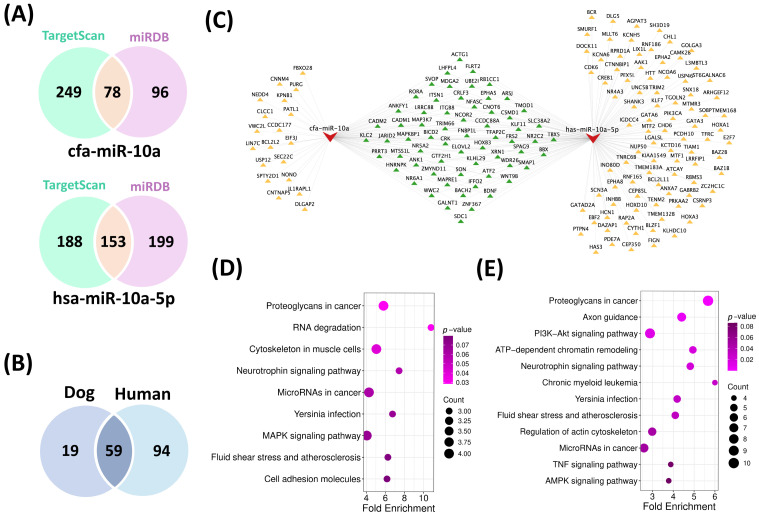
Comparative target gene analysis and functional enrichment of cfa-miR-10a and its human ortholog. (**A**) Venn diagram showing common target genes for cfa-miR-10a and hsa-miR-10a-5p predicted using TargetScan and miRDB. (**B**) Overlapping target genes shared between dog and human. (**C**) miRNA-mRNA interaction network constructed in Cytoscape (v3.10.3), displaying 59 shared target genes between cfa-miR-10a and hsa-miR-10a-5p. The red node represents miRNAs, yellow nodes indicate unique targets of each miRNA, and green nodes represent shared targets between the species (**D**) KEGG pathway enrichment for cfa-miR-10a targets (**E**) KEGG pathway enrichment for hsa-miR-10a-5p targets. The KEGG pathways are listed on the left, while *p*-values and gene counts are shown on the right. Pathways with a *p*-value < 0.05 were considered significantly enriched.

Target genes were predicted for canine miR-10a (cfa-miR-10a) and its conserved human counterpart hsa-miR-10a-5p using TargetScan and miRDB (Supplementary Table S4), yielding 78 common targets in dog and 153 in human (Figure 4A). Subsequent Venn diagram analysis revealed 59 overlapping target genes shared between the two species (Figure 4B). The corresponding miRNA-target interaction network was constructed and visualized using Cytoscape v3.10.3 (Figure 4C).

Functional enrichment analysis of these targets using the DAVID platform revealed 22 significantly (*p* < 0.05) enriched GO terms for cfa-miR-10a (Supplementary Table S5) and 85 terms for hsa-miR-10a-5p (Supplementary Table S6) across the three categories (BP, CC and MF). Within the biological process (BP) category, shared terms included regulation of transcription by RNA polymerase II, liver development, and cell migration, while in the molecular function (MF) category, common terms included RNA polymerase II-specific DNA-binding transcription factor activity, DNA binding, nuclear receptor activity, and protein binding. KEGG analysis identified three significant (*p* < 0.05) pathways for cfa-miR-10a (Supplementary Table S7) and 15 pathways (*p* < 0.05) for hsa-miR-10a-5p (Supplementary Table S8). Notably, only one pathway, Proteoglycans in cancer (*p* < 0.05), was conserved across both species, with ACTG1, SDC1, FRS2, and WNT9B identified as common genes participating in this pathway (Figure 4D,E; Supplementary Tables S7 and S8). Among these four target genes, expression analysis using the GEPIA and UALCAN databases revealed that ACTG1 was consistently and strongly upregulated in HCC cases (Supplementary Figures S3 and S4).

## 4. Discussion

HCC is increasingly well characterized at the molecular level, but much remains to be studied in relation to the role of specific miRNAs, especially in the canine disease. In this study, we focused on cfa-miR-10a, evaluating its expression profile and biomarker potential across multiple biological compartments (tumor tissue, plasma, and plasma-derived exosomes), and we then investigated the expression of its human ortholog in human HCC. Our findings demonstrate a divergent expression pattern of miR-10a in canine HCC, with consistent downregulation in tumor tissues but contrasting trends in circulating compartments, while comparative analyses in human HCC further underscore species-specific and sample-type-dependent differences.

In the present study, cfa-miR-10a was significantly downregulated in canine HCC tissues compared with normal liver controls, suggesting a potential tumor-suppressive role in canine liver cancer. To the best of our knowledge, no previous studies have reported cfa-miR-10a expression patterns in canine HCC, and only limited information is available for other canine cancers. Similarly to the present study, cfa-miR-10a was downregulated (fold change: −2.5) in canine diffuse large B-cell lymphoma compared to healthy lymph node [39]. Given the limited data on canine HCC, we compared our findings with human studies. Online TCGA databases (ENCORI and UALCAN) confirmed downregulation of hsa-miR-10a-5p in human HCC tissues, consistent with previous reports [25,31]. Beyond HCC, hsa-miR-10a-5p has also been reported as downregulated in several human cancers such as chronic myeloid leukemia [26], melanoma [40], prostate cancer [41], ovarian cancer [42,43], and cervical cancer [27]. Conversely, miR-10a-5p is upregulated in other human cancers including cholangiocarcinoma [28], breast [29], colorectal [30], bladder [44], lung [45], and pancreatic cancers [46], supporting its dual roles [24], acting either as an oncomiR or tumor suppressor miRNA (TSmiR) depending on tissue context. Our canine HCC data are consistent with the tumor-suppressive pattern, suggesting that loss of miR-10a may derepress oncogenic targets and promote hepatocellular carcinogenesis. Notably, even within HCC, miR-10a can exhibit context-dependent functionality, modulating tumor progression depending on downstream targets [24,25,47].

Plasma-derived miR-10a levels in canine HCC patients did not differ significantly from healthy controls, in contrast to the obvious tissue-specific downregulation. Findings in the plasma from the present study contrast with the study conducted by Shen et al. (2021) where miR-10a was downregulated in plasma of HCC patients [25]. As circulating free miRNAs are highly susceptible to RNases degradation [19,48,49], we investigated the expression of cfa-miR-10a in plasma exosomes, which provide a protective lipid bilayer that enhances RNA stability compared to freely circulating RNAs in plasma. Strikingly, when focusing on plasma-derived exosomes, we observed a significant upregulation of cfa-miR-10a in HCC patients versus healthy animals, with high diagnostic accuracy (AUC = 0.94) demonstrated by ROC curve analysis, highlighting the importance of exosomes as a more reliable and enriched source of tumor-derived miRNAs. The exosomal high expression of miR-10a in canine HCC was coincided with the high expression of hsa-miR-10a-5p in the circulatory compartment of human HCC [50].

The opposite pattern of expression patterns of miR-10a in canine HCC tissue (downregulation) and the circulatory compartment (upregulation) highlight the heterogeneity of miRNA profiles between tumor cells and circulation. This finding aligns with the report by Bai et al. (2019), which described a similar divergent expression pattern of hsa-miR-10a-5p in human HCC tissue and plasma [50]. Similar discrepancies between tissue and circulating exosomal miRNA levels have been reported in other types of tumors as well. For example, miR-454-3p was found to be significantly downregulated in glioma tumor tissues but markedly upregulated in exosomes from the same patients [22]. Similarly, miR-1228-3p was reported to be downregulated in colorectal tissues while showed upregulation in patient’s plasma [21]. However, the observed intriguing outcome of upregulation of miR-10a in exosomes, despite its downregulation in tumor tissues, suggests an active export mechanism whereby tumor cells may selectively secrete specific miRNAs into the extracellular environment to rescue cells from intracellular tumor-suppressive pressure resulting in differential miRNA expression profile in bodily fluid versus the original tissue source [21,22]. In addition to selective export of miRNAs by cancer cells, other cells, such as the circulating blood cells, may also secrete miRNAs into the plasma, contributing to elevated circulating levels despite their downregulation in the original tumor tissue [50]. This phenomenon warrants further in vitro studies using cancer specific cell lines to elucidate the mechanisms underlying the increased expression in circulation.

Similarly to our observation with cfa-miR-10a, where no significant change was detected in patient plasma but a marked upregulation was noticed in exosomes, compartment-specific difference in miRNA abundance has also been reported in other setting. For example, miR-21 displayed opposing expression patterns in paired serum and plasma samples from patients with acute myocardial infarction [51]. This underscores that biofluid type and vesicle association can markedly influence detectable miRNA levels.

Due to the lack of published data on the expression of miR-10a in specifically the exosomal part of human HCC, we downloaded a publicly available human HCC datasets (GSE83977) focusing on serum exosomal miRNAs. Unlike the upregulated expression in canine exosomal miRNAs, hsa-miR-10a showed a non-significant downregulation in human HCC serum exosomes. These results suggest a partially conserved yet species- and sample-dependent pattern of miR-10a regulation. This discrepant expression of miR-10a in the circulating compartment between canine and human HCC suggest differences in underlying regulatory mechanisms. In canine HCC, the significant upregulation of miR-10a in plasma exosomes, despite its downregulation in tissues, supports the possibility of selective and active export of miRNAs into extracellular vesicles [21,22]. In contrast, in human HCC, although hsa-miR-10a-5p (the human homolog of cfa-miR-10a) is downregulated in tissue, it is not significantly altered in plasma exosomes. This could indicate reduced overall production of the miRNA, miRNA sponging by competing endogenous RNAs [52], or a lack of efficient exosomal export, leading to increase free RNA in the circulation followed by the degradation of circulatory RNases [49]. Together, these observations suggest that certain miRNAs may exhibit species-specific differences in biogenesis, regulation, and vesicular export, which may underlie the divergent tissue and circulating profiles of miR-10a observed between canine and human HCC.

Cross-species analysis of miR-10a target genes revealed both species-specific and conserved regulatory networks. Notably, the KEGG pathway “Proteoglycans in cancer” emerged as the only pathway significantly enriched in both canine and human target gene sets, suggesting a conserved role of miR-10a in modulating tumor-associated proteoglycan signaling. Proteoglycans are well-established regulators of hepatocellular carcinoma progression, influencing cell proliferation, migration, and extracellular matrix (ECM) interactions [53]. ACTG1, SDC1, FRS2, and WNT9B were commonly identified as genes contributing to the enrichment of the ‘Proteoglycans in Cancer’ pathway in both species. Moreover, ACTG1, previously identified as a target of miR-10a in colorectal cancer [30], was also found to be strongly upregulated in human HCC cells [54]. Given that the miR-10a/ACTG1 regulatory axis may contribute to the enrichment of the ‘Proteoglycans in Cancer’ pathway, it could play a role in cross-species HCC progression.

From a translational perspective, our findings support the potential utility of exosomal miR-10a as a minimally invasive biomarker for canine HCC. The high diagnostic performance observed in ROC curve analysis suggests that exosomal cfa-miR-10a could aid in early detection or disease monitoring, complementing tissue biopsy-based methods. However, the discrepancy between canine and human exosome datasets indicates that its biomarker applicability in human HCC remains limited or context-dependent. This highlights the need for larger validation cohorts, standardized methodologies, and integrated multi-omics approaches to confirm the clinical utility of miR-10a across species.

Several limitations should be acknowledged. One of the limitations of this study is the small sample size used for NGS analysis (*n* = 3 per group). Although the NGS sample size was small, subsequent RT-qPCR validation across independent samples strengthened the reliability of the findings. Additionally, detailed physiological and clinical parameters for some samples were unavailable, as the samples had been collected several years ago and the associated clinical records were incomplete or poorly documented. Moreover, the functional role of miR-10a in HCC biology remains unclear, and its predicted target genes (ACTG1, SDC1, FRS2, and WNT9B) were not clinically validated. Therefore, larger cohort with different breeds, and disease stages are warranted to validate these targets and elucidate the underlying regulatory mechanisms. The mechanisms governing the exosomal packaging and secretion of cfa-miR-10a were not elucidated in this study. Furthermore, the cellular origin of its elevated exosomal levels remains to be determined, as the absence of a normal canine hepatocyte cell line limited our ability to assess the direct contribution of tumor cells to the increased circulating cfa-miR-10a observed in canine HCC. Future studies using larger cohorts and mechanistic assays are needed to elucidate these aspects.

## 5. Conclusions

In conclusion, our study provides novel insights into the divergent expression patterns of cfa-miR-10a in canine hepatocellular carcinoma (HCC) across tissue, plasma, and exosomal compartments, with comparative validation in human HCC dataset. In both species, miR-10a was consistently downregulated in tumor tissues, supporting its potential conserved tumor-suppressive role. However, contrasting trends were observed in the circulatory compartment: while miR-10a was enriched in canine plasma exosomes, suggesting selective export, its expression was reduced in human plasma and plasma exosome. This cross-species contrast suggests that miR-10a trafficking and vesicular export may follow species-specific regulatory mechanisms, underscoring the importance of comparative oncology approaches to unravel conserved versus divergent features of miRNA biology in HCC. Collectively, our findings highlight the complexity of miRNA regulation in cancer and reinforce the translational potential of exosome-derived miRNAs as minimally invasive biomarkers. Further validation in larger canine cohorts and mechanistic studies exploring miRNA trafficking are warranted to clarify the functional significance of miR-10a and to evaluate its clinical applicability in the diagnosis and management of HCC.

## Data Availability

The original contributions presented in this study are included in the article/Supplementary Material. The analyzed data underlying this study are openly available in Figshare at DOI: 10.6084/m9.figshare.30075649, accessed on 22 September 2025, Serum exosomal RNA-seq data of human HCC presented in this study are available in the Gene Expression Omnibus (GEO) database at https://www.ncbi.nlm.nih.gov/geo/query/acc.cgi?acc=GSE83977, accessed on 20 March 2025, reference number GSE83977. The canine HCC datasets analyzed in this study are available in the NCBI BioProject database at https://www.ncbi.nlm.nih.gov/bioproject/?term=PRJNA1293103, accessed on 18 July 2025, reference number PRJNA1293103.

## Supplementary Materials

The following supporting information can be downloaded at https://www.mdpi.com/article/10.3390/cimb47110950/s1.

## Abbreviations

The following abbreviations are used in this manuscript:

HCC	Hepatocellular carcinoma
AUC	Area under the curve
TCGA	The cancer genome atlas
KEGG	Kyoto encyclopedia of genes and genomes
KUVTH	Kagoshima university veterinary teaching hospital
PBS	Phosphate-buffered saline
NGS	Next generation sequencing
cDNA	Complementary DNA
DE	Differentially expressed
FDR	False discovery rate
PCA	Principal component analysis
RT-qPCR	Reverse transcription–quantitative polymerase chain reaction.
SDS-PAGE	Sodium dodecyl sulfate–polyacrylamide gel electrophoresis
PVDF	Polyvinylidene difluoride
TBST	Tris-buffered saline with tween 20
ENCORI	Encyclopedia of RNA interactomes
GEPIA	Gene expression profiling interactive analysis
UALCAN	The university of Alabama at Birmingham cancer data analysis portal
GEO	Gene expression omnibus
GO	Gene ontology
ROC	Receiver operating characteristic
BP	Biological process
CC	Cellular component
MF	Molecular function
TSmiR	Tumor suppressor miRNA
