# Supplementary material for: Divergent Tissue and Circulatory Expression of miR-10a in Canine Hepatocellular Carcinoma: Comparative Insights from Human HCC
Source: Curr Issues Mol Biol. 2025 Nov 15;47(11):950. doi: 10.3390/cimb47110950 (PMC12651625; doi:10.3390/cimb47110950)
Supplement: Supplementary file 1 [file cimb-47-00950-s001.zip › cimb-3953883-supplementary/Suppl_Files/Supplementary Materials 1.pdf]

# Divergent Tissue and Circulatory Expression of miR-10a in Canine Hepatocellular Carcinoma: Comparative Insights from Human HCC

**Most Shumi Akhter Shathi [1], Mohammad Arif [1,3], Nobuhiro Nozaki [1], Yutaro Ide [1], Yoshiyuki Akiyama [1], Shaohsu Wang [1], Osamu Yamato [1] and Naoki Miura [1,2,*]**

1 Joint Graduate School of Veterinary Medicine, Kagoshima University, Japan
2 Veterinary Teaching Hospital, Joint Faculty of Veterinary Medicine, Kagoshima University, Japan
3 Department of Microbiology and Hygiene, Bangladesh Agricultural University, Mymensingh-2202, Bangladesh
* Correspondence: Naoki Miura, Joint Graduate School of Veterinary Medicine, Kagoshima University, Japan, k9236024@kadai.jp; Tel.: +81-99-285-3527

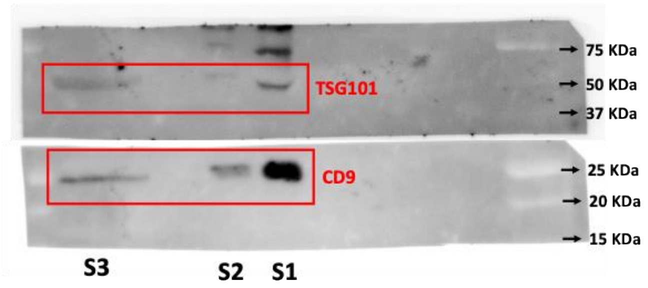


**Supplementary Figure S1: Full western blot image showing specific bands for the exosomal markers CD9 and TSG101.** S1, S2 and S3 correspond to three independent exosome preparations. Molecular weight markers (kDa) are indicated on the right side.

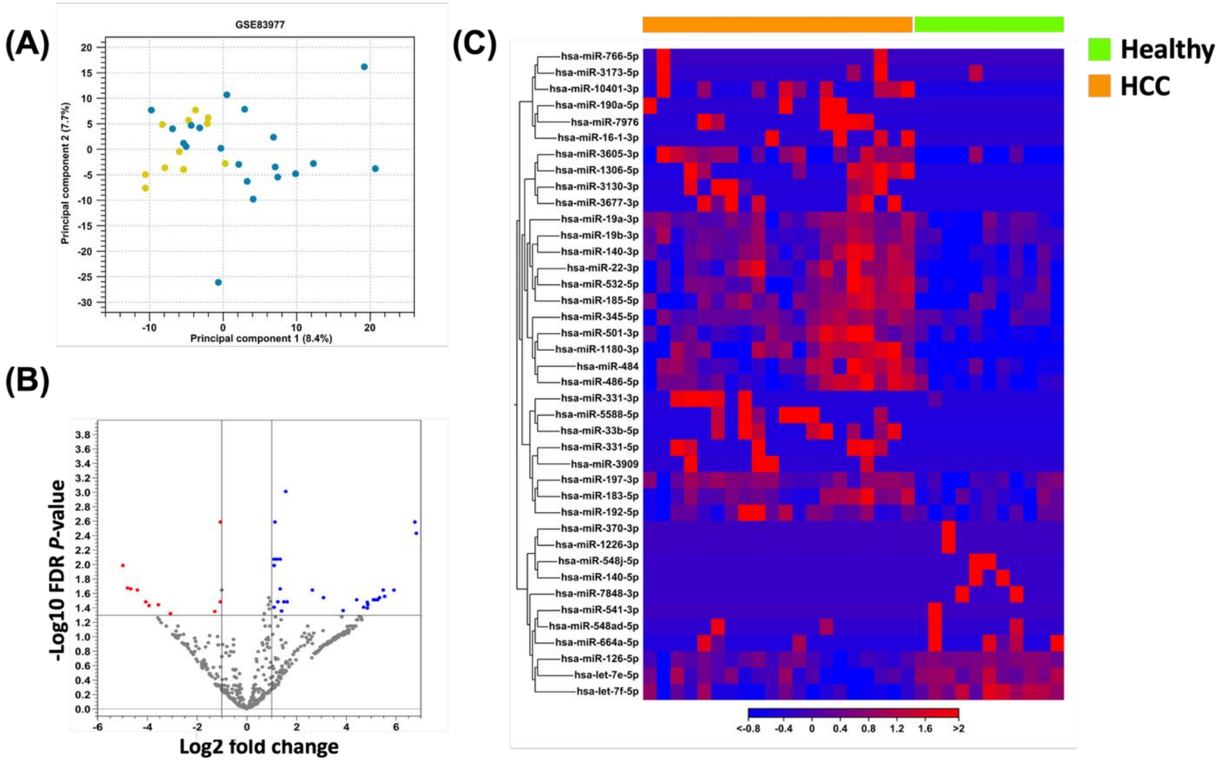


**Supplementary Figure S2: Transcriptomic overview of differentially expressed serum exosomal miRNAs in human HCC from the GEO dataset (GSE83977).** (A) PCA plot showing the clustering of HCC serum exosomes and healthy serum exosomes. Yellow dots represent healthy exosomes, and blue dots represent HCC exosomes. (B) Volcano plot displaying differentially expressed miRNAs. Blue dots represent upregulated miRNAs, red dots represent downregulated miRNAs, and gray dots represent miRNAs that were not differentially expressed. Differentially expressed miRNAs were identified using the criteria |fold change| > 2 and FDR-adjusted *p*-value < 0.05. (C) Hierarchical clustering of differentially expressed exosome-derived miRNAs in human HCC based on their expression profiles.

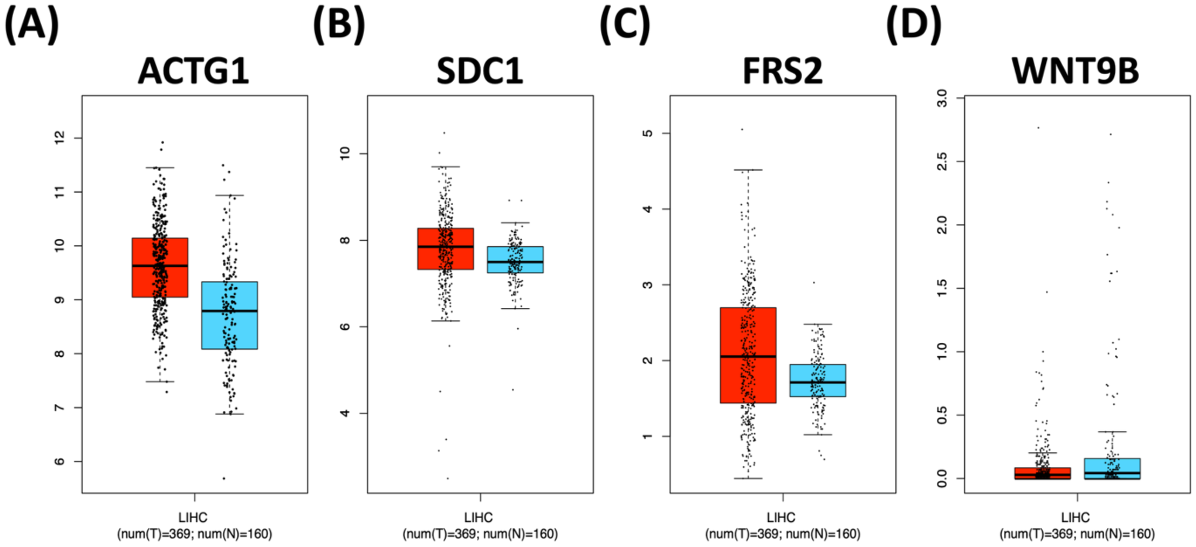


**Supplementary Figure S3: Expression analysis of target genes contributing to the enrichment of the Proteoglycans in cancer pathway in canine and human HCC using the GEPIA2 online database.** Expression levels of ACTG1 (A), SDC1 (B), FRS2 (C), and WNT9B (D) in HCC tissues. Red box represents tumor tissues and blue one represents normal liver tissues.

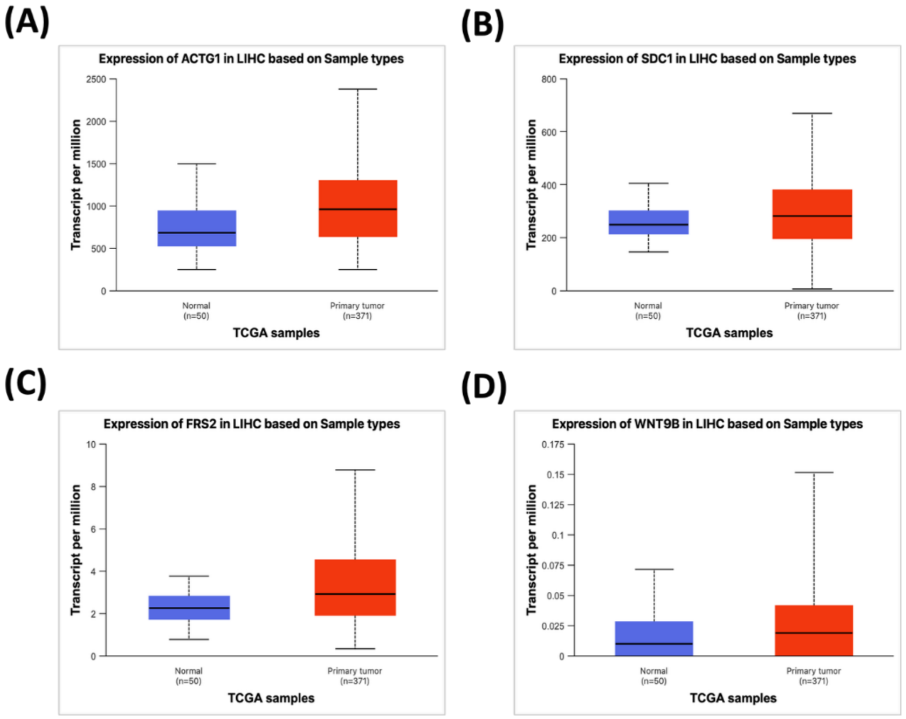


**Supplementary Figure S4: Expression analysis of target genes contributing to the enrichment of the Proteoglycans in Cancer pathway in canine and human HCC using the UALCAN online database.** Expression levels of ACTG1 (A), SDC1 (B), FRS2 (C), and WNT9B (D) in HCC tissues. Red box represents tumor tissues and blue one represents normal liver tissues.

**Supplementary Table S1: Details of samples included in this study**

| Samples | Age | Diagnosis | Sex | Spayed/ Neutered | Breed | Tissue | Blood |
|---|---|---|---|---|---|---|---|
| NL-1 | - | Healthy | - | - | Beagle | 6 | - |
| NL-2 | - | Healthy | - | - | Beagle | | |
| NL-3 | - | Healthy | - | - | Beagle | | |
| NL-4 | - | Healthy | - | - | Beagle | | |
| NL-5 | - | Healthy | - | - | Beagle | | |
| NL-6 | - | Healthy | - | - | Beagle | | |
| HB-1 | 4 Y 8 M | Healthy | M | No | Toy Poodle | - | 7 |
| HB-2 | 5 Y 3 M | Healthy | M | Yes | Mong | | |
| HB-3 | 5 Y 4 M | Healthy | M | No | Pomeranian | | |
| HB-4 | 7 Y 5 M | Healthy | F | Yes | Toy Poddle | | |
| HB-5 | 11 Y 2 M | Healthy | F | No | Chihuahua | | |
| HB-6 | 6 Y 4 M | Healthy | F | No | Shiba | | |
| HB-7 | 7 Y | Healthy | F | Yes | Mong | | |
| P1 | 11 Y 7 M | HCC | F | Yes | Shiba Inu | 14 | 14 |
| P2 | 11 Y 10 M | HCC | F | Yes | Shiba Inu | | |
| P3 | 14 Y | HCC | F | Yes | Crossbreed | | |
| P4 | 10 Y 9 M | HCC | F | No | Crossbreed | | |
| P5 | 10 Y 3 M | HCC | F | No | Beagle | | |
| P6 | 10 Y 9 M | HCC | F | No | Yorkshire Terrier | | |
| P7 | 12 Y | HCC | F | No | Miniature Schnauzer | | |
| P8 | 13 Y 10 M | HCC | F | No | Shetland Sheepdog | | |
| P9 | 12 Y 3 M | HCC | F | No | Chihuahua | | |
| P10 | 10 Y 8 M | HCC | M | Yes | Shiba | | |
| P11 | 11 Y 7 M | HCC | M | Yes | Welsh Corgi | | |
| P12 | 11 Y 6 M | HCC | M | No | Shiba | | |
| P13 | 11 Y 10 M | HCC | M | No | Yorkshire Terrier | | |
| P14 | 10 Y 4 M | HCC | M | Yes | Crossbreed | | |

NL - Normal liver tissue from experimental animal at Shin Nippon Biomedical Laboratories (SNBL, Kagoshima, Japan); HB- Blood samples from apparently healthy dogs collected from Chuou Aiken Animal Hospital, Kagoshima, Japan; P – Patient; Y – Year; M – Month; HCC - Hepatocellular carcinoma

**Supplementary Table S2: A list of differentially expressed miRNAs in canine HCC.** Following criteria were applied to filter the differentially expressed miRNAs: |fold change| > 2, FDR *p*-value < 0.05, and maximum group mean > 10.

| Differential expression (No.) | Name | Max group mean | $Log_2$ fold change | Fold change | *p*-value | FDR *p*-value | Bonferroni |
|---|---|---|---|---|---|---|---|
| **Upregulated (46)** | hsa-let-7i-5p | 273,077.33 | 2 | 4.01 | 2.46E-03 | 0.03 | 1 |
| | cfa-miR-222 | 47,745.33 | 2.36 | 5.13 | 1.39E-03 | 0.02 | 1 |
| | cfa-miR-182 | 32,497.67 | 3.32 | 9.96 | 1.05E-06 | 6.39E-05 | 7.97E-04 |
| | hsa-miR-193a-3p | 30,260.33 | 2.55 | 5.88 | 4.46E-04 | 8.77E-03 | 0.34 |
| | cfa-miR-301a | 18,187.67 | 1.8 | 3.47 | 2.63E-03 | 0.03 | 1 |
| | cfa-miR-8908a-3p | 15,266.33 | 11.76 | 3,465.02 | 2.39E-15 | 1.45E-12 | 1.81E-12 |
| | cfa-miR-34a | 8,445.33 | 3.15 | 8.88 | 3.58E-09 | 5.45E-07 | 2.72E-06 |
| | cfa-miR-483 | 5,589.67 | 4.26 | 19.11 | 9.52E-04 | 0.02 | 0.72 |
| | cfa-miR-183 | 2,248.33 | 2.89 | 7.44 | 3.13E-05 | 9.53E-04 | 0.02 |
| | hsa-let-7i-3p | 1,867.00 | 2.08 | 4.22 | 2.22E-03 | 0.03 | 1 |
| | hsa-miR-210-3p | 1,595.00 | 3.03 | 8.16 | 3.36E-05 | 9.73E-04 | 0.03 |
| | cfa-miR-551a | 1,204.67 | 2.78 | 6.85 | 2.59E-04 | 5.43E-03 | 0.2 |
| | cfa-miR-184 | 1,076.67 | 7.95 | 248.03 | 1.94E-09 | 3.94E-07 | 1.47E-06 |
| | hsa-miR-675-3p | 783.33 | 6.29 | 78 | 8.78E-08 | 1.07E-05 | 6.67E-05 |
| | cfa-miR-8908b | 760.67 | 12.06 | 4,263.99 | 6.34E-06 | 2.52E-04 | 4.82E-03 |
| | hsa-miR-5701 | 609 | 2.94 | 7.7 | 6.52E-04 | 0.01 | 0.5 |
| | cfa-miR-135b | 602.67 | 5.4 | 42.18 | 2.62E-05 | 8.87E-04 | 0.02 |
| | hsa-miR-105-5p | 596 | 5.54 | 46.38 | 1.39E-06 | 7.72E-05 | 1.06E-03 |
| | cfa-miR-8865 | 353.33 | 5.35 | 40.72 | 1.25E-09 | 3.80E-07 | 9.50E-07 |
| | cfa-miR-8908c | 344 | 10.91 | 1,928.36 | 5.89E-05 | 1.50E-03 | 0.04 |
| | cfa-miR-8908d | 341.33 | 10.9 | 1,913.42 | 5.92E-05 | 1.50E-03 | 0.04 |
| | cfa-miR-210 | 322.67 | 3.49 | 11.25 | 9.44E-04 | 0.02 | 0.72 |
| | cfa-miR-6529 | 222.67 | 3.03 | 8.19 | 2.95E-03 | 0.03 | 1 |
| | hsa-miR-105-3p | 208.33 | 5.1 | 34.31 | 1.42E-05 | 5.08E-04 | 0.01 |
| | hsa-miR-1271-3p | 177.33 | 5.12 | 34.87 | 1.00E-06 | 6.39E-05 | 7.60E-04 |
| | cfa-miR-96 | 172.33 | 2.56 | 5.89 | 5.85E-04 | 0.01 | 0.44 |
| | hsa-miR-1224-5p | 171 | 2.91 | 7.51 | 9.98E-04 | 0.02 | 0.76 |
| | cfa-miR-454 | 166.67 | 2.08 | 4.23 | 1.77E-03 | 0.02 | 1 |
| | hsa-miR-10395-3p | 166 | 2.8 | 6.99 | 1.17E-03 | 0.02 | 0.89 |
| | hsa-miR-877-5p | 163 | 3.42 | 10.67 | 2.49E-04 | 5.41E-03 | 0.19 |
| | hsa-miR-34a-3p | 146.67 | 3.85 | 14.45 | 6.63E-06 | 2.52E-04 | 5.04E-03 |
| | cfa-miR-105a | 142 | 4.81 | 27.96 | 1.35E-04 | 3.16E-03 | 0.1 |
| | cfa-miR-8908a-5p | 127.33 | 9.48 | 714.49 | 3.15E-04 | 6.39E-03 | 0.24 |
| | hsa-miR-4508 | 85 | 2.94 | 7.65 | 2.36E-04 | 5.33E-03 | 0.18 |

| | | | | | | | |
|---|---|---|---|---|---|---|---|
| | hsa-miR-7975 | 65.33 | 3.15 | 8.9 | 1.39E-03 | 0.02 | 1 |
| | cfa-miR-2387 | 65 | 2.48 | 5.6 | 3.01E-03 | 0.03 | 1 |
| | hsa-miR-190a-3p | 60.67 | 2.46 | 5.5 | 1.84E-03 | 0.02 | 1 |
| | hsa-miR-4443 | 59.33 | 3.49 | 11.21 | 4.62E-05 | 1.28E-03 | 0.04 |
| | hsa-miR-4492 | 45 | 3.74 | 13.33 | 4.05E-06 | 1.89E-04 | 3.07E-03 |
| | cfa-miR-551b | 38 | 2.7 | 6.51 | 2.07E-03 | 0.03 | 1 |
| | cfa-miR-105b | 30.33 | 5.61 | 48.96 | 1.18E-03 | 0.02 | 0.9 |
| | hsa-miR-302b-3p | 27 | 4.51 | 22.78 | 1.10E-03 | 0.02 | 0.83 |
| | cfa-miR-196a | 20.33 | 4.53 | 23.07 | 1.85E-03 | 0.02 | 1 |
| | cfa-miR-205 | 19 | 3.43 | 10.8 | 3.89E-03 | 0.04 | 1 |
| | hsa-miR-4485-3p | 18.67 | 7.07 | 134.53 | 4.02E-03 | 0.04 | 1 |
| | hsa-miR-183-3p | 14.33 | 4.81 | 28.06 | 1.21E-03 | 0.02 | 0.92 |
| **Downregulated (13)** | **cfa-miR-10a** | **1,629,083.33** | **-2.43** | **-5.37** | **5.20E-04** | **9.91E-03** | **0.4** |
| | hsa-miR-30a-5p | 961,197.00 | -1.93 | -3.81 | 1.32E-03 | 0.02 | 1 |
| | cfa-miR-375 | 108,425.33 | -3.54 | -11.61 | 2.30E-06 | 1.17E-04 | 1.75E-03 |
| | cfa-miR-146a | 36,973.67 | -3.16 | -8.95 | 8.19E-07 | 6.24E-05 | 6.23E-04 |
| | cfa-miR-215 | 3,816.67 | -2.65 | -6.27 | 4.87E-06 | 2.12E-04 | 3.70E-03 |
| | cfa-miR-214 | 3,796.33 | -1.88 | -3.67 | 2.77E-03 | 0.03 | 1 |
| | cfa-miR-338 | 1,401.00 | -2.63 | -6.2 | 2.88E-05 | 9.23E-04 | 0.02 |
| | hsa-miR-338-3p | 1,058.67 | -2.37 | -5.15 | 1.32E-03 | 0.02 | 1 |
| | cfa-miR-490 | 437.33 | -4.24 | -18.96 | 4.62E-07 | 4.02E-05 | 3.51E-04 |
| | hsa-miR-590-5p | 69.67 | -7.42 | -170.8 | 2.00E-07 | 2.03E-05 | 1.52E-04 |
| | hsa-miR-1-3p | 58.67 | -3.45 | -10.92 | 1.35E-04 | 3.16E-03 | 0.1 |
| | cfa-miR-1 | 16 | -3.28 | -9.73 | 4.23E-03 | 0.04 | 1 |
| | hsa-miR-323b-3p | 12.33 | -4.9 | -29.91 | 1.34E-03 | 0.02 | 1 |

**Supplementary Table S3: A list of differentially expressed exosomal miRNAs in human HCC using GSE83977 dataset.** Following criteria were applied to filter the differentially expressed miRNAs: |fold change| > 2, and FDR *p*-value < 0.05.

| Name | Max group mean | $Log_2$ fold change | Fold change | *p*-value | FDR *p*-value | Bonferroni |
|---|---|---|---|---|---|---|
| hsa-miR-486-5p | 1.15E+07 | 1.11 | 2.16 | 1.76E-04 | 0.01 | 0.13 |
| hsa-let-7f-5p | 19,246.82 | -1.05 | -2.07 | 1.32E-05 | 2.59E-03 | 9.81E-03 |
| hsa-miR-22-3p | 9,907.45 | 1.14 | 2.2 | 1.77E-05 | 2.60E-03 | 0.01 |
| hsa-miR-192-5p | 7,255.65 | 1.11 | 2.16 | 2.55E-03 | 0.04 | 1 |
| hsa-miR-126-5p | 1,970.09 | -1.06 | -2.09 | 1.77E-03 | 0.03 | 1 |
| hsa-miR-501-3p | 1,148.70 | 1.57 | 2.96 | 1.66E-06 | 9.76E-04 | 1.23E-03 |
| hsa-miR-484 | 523.95 | 1.1 | 2.14 | 1.02E-04 | 8.50E-03 | 0.08 |
| hsa-miR-19b-3p | 444.15 | 1.35 | 2.55 | 4.81E-04 | 0.02 | 0.36 |
| hsa-miR-532-5p | 410.85 | 1.35 | 2.55 | 1.09E-04 | 8.50E-03 | 0.08 |
| hsa-miR-140-3p | 348.1 | 1.21 | 2.31 | 1.16E-04 | 8.50E-03 | 0.09 |
| hsa-miR-197-3p | 267.9 | 1.62 | 3.07 | 1.60E-03 | 0.03 | 1 |
| hsa-miR-183-5p | 208.3 | 1.49 | 2.81 | 1.79E-03 | 0.03 | 1 |
| hsa-miR-19a-3p | 197.75 | 1.25 | 2.38 | 1.71E-03 | 0.03 | 1 |
| hsa-let-7e-5p | 167.64 | -1.28 | -2.42 | 3.38E-03 | 0.05 | 1 |
| hsa-miR-345-5p | 58.7 | 1.39 | 2.63 | 3.25E-03 | 0.04 | 1 |
| hsa-miR-185-5p | 19.95 | 2.65 | 6.26 | 5.50E-04 | 0.02 | 0.41 |
| hsa-miR-664a-5p | 12.55 | -3.07 | -8.37 | 3.67E-03 | 0.05 | 1 |
| hsa-miR-3605-3p | 11.1 | 3.08 | 8.48 | 1.04E-03 | 0.03 | 0.77 |
| hsa-miR-548ad-5p | 8.55 | -3.55 | -11.74 | 2.18E-03 | 0.04 | 1 |
| hsa-miR-10401-3p | 7.05 | 6.82 | 113.01 | 3.18E-05 | 3.74E-03 | 0.02 |
| hsa-miR-1180-3p | 6.5 | 6.78 | 109.69 | 1.22E-05 | 2.59E-03 | 9.03E-03 |
| hsa-miR-541-3p | 6.18 | -4.8 | -27.8 | 4.00E-04 | 0.02 | 0.3 |
| hsa-miR-548j-5p | 5.09 | -4.06 | -16.64 | 1.68E-03 | 0.03 | 1 |
| hsa-miR-7848-3p | 4.91 | -4.98 | -31.57 | 1.73E-04 | 0.01 | 0.13 |
| hsa-miR-190a-5p | 4.4 | 5.56 | 47.07 | 9.00E-04 | 0.03 | 0.67 |
| hsa-miR-3173-5p | 4.25 | 3.88 | 14.75 | 3.14E-03 | 0.04 | 1 |
| hsa-miR-1226-3p | 4 | -4.66 | -25.32 | 4.82E-04 | 0.02 | 0.36 |
| hsa-miR-5588-5p | 3.7 | 5.49 | 44.98 | 6.35E-04 | 0.02 | 0.47 |
| hsa-miR-766-5p | 3.65 | 5.92 | 60.7 | 6.32E-04 | 0.02 | 0.47 |
| hsa-miR-140-5p | 3.45 | -4.39 | -21.02 | 6.97E-04 | 0.02 | 0.52 |
| hsa-miR-3130-3p | 3.4 | 5.34 | 40.5 | 1.02E-03 | 0.03 | 0.75 |
| hsa-miR-331-3p | 3.25 | 4.43 | 21.56 | 1.24E-03 | 0.03 | 0.92 |
| hsa-miR-370-3p | 2.36 | -3.93 | -15.23 | 2.34E-03 | 0.04 | 1 |
| hsa-miR-3677-3p | 2.35 | 5.1 | 34.22 | 1.37E-03 | 0.03 | 1 |
| hsa-miR-3909 | 2.25 | 4.86 | 29.03 | 2.73E-03 | 0.04 | 1 |
| hsa-miR-16-1-3p | 2.15 | 5.26 | 38.45 | 1.37E-03 | 0.03 | 1 |

| | | | | | | |
|---|---|---|---|---|---|---|
| hsa-miR-7976 | 2.05 | 5.18 | 36.23 | 1.32E-03 | 0.03 | 0.98 |
| hsa-miR-33b-5p | 1.9 | 4.85 | 28.76 | 1.88E-03 | 0.03 | 1 |
| hsa-miR-331-5p | 1.65 | 4.86 | 29.14 | 2.23E-03 | 0.04 | 1 |
| hsa-miR-1306-5p | 1.5 | 4.7 | 25.93 | 2.61E-03 | 0.04 | 1 |
